# Effects of Biliverdin Administration on Acute Lung Injury Induced by Hemorrhagic Shock and Resuscitation in Rats

**DOI:** 10.1371/journal.pone.0063606

**Published:** 2013-05-07

**Authors:** Junko Kosaka, Hiroshi Morimatsu, Toru Takahashi, Hiroko Shimizu, Susumu Kawanishi, Emiko Omori, Yasumasa Endo, Naofumi Tamaki, Manabu Morita, Kiyoshi Morita

**Affiliations:** 1 Department of Anesthesiology and Resuscitology, Okayama University Graduate School of Medicine, Dentistry and Pharmaceutical Sciences, Okayama, Japan; 2 Faculty of Health and Welfare Science, Okayama Prefectural University, Okayama, Japan; 3 Department of Preventive Dentistry, Okayama University Graduate School of Medicine, Dentistry and Pharmaceutical Sciences, Okayama, Japan; 4 Department of Preventive Dentistry, Institute of Health Biosciences, The University of Tokushima Graduate School, Tokushima, Japan; UAE University, Faculty of Medicine & Health Sciences, United Arab Emirates

## Abstract

Hemorrhagic shock and resuscitation induces pulmonary inflammation that leads to acute lung injury. Biliverdin, a metabolite of heme catabolism, has been shown to have potent cytoprotective, anti-inflammatory, and anti-oxidant effects. This study aimed to examine the effects of intravenous biliverdin administration on lung injury induced by hemorrhagic shock and resuscitation in rats. Biliverdin or vehicle was administered to the rats 1 h before sham or hemorrhagic shock-inducing surgery. The sham-operated rats underwent all surgical procedures except bleeding. To induce hemorrhagic shock, rats were bled to achieve a mean arterial pressure of 30 mmHg that was maintained for 60 min, followed by resuscitation with shed blood. Histopathological changes in the lungs were evaluated by histopathological scoring analysis. Inflammatory gene expression was determined by Northern blot analysis, and oxidative DNA damage was assessed by measuring 8-hydroxy-2′ deoxyguanosine levels in the lungs. Hemorrhagic shock and resuscitation resulted in prominent histopathological damage, including congestion, edema, cellular infiltration, and hemorrhage. Biliverdin administration prior to hemorrhagic shock and resuscitation significantly ameliorated these lung injuries as judged by histopathological improvement. After hemorrhagic shock and resuscitation, inflammatory gene expression of tumor necrosis factor-α and inducible nitric oxide synthase were increased by 18- and 8-fold, respectively. Inflammatory gene expression significantly decreased when biliverdin was administered prior to hemorrhagic shock and resuscitation. Moreover, after hemorrhagic shock and resuscitation, lung 8-hydroxy-2' deoxyguanosine levels in mitochondrial DNA expressed in the pulmonary interstitium increased by 1.5-fold. Biliverdin administration prior to hemorrhagic shock and resuscitation decreased mitochondrial 8-hydroxy-2' deoxyguanosine levels to almost the same level as that in the control animals. We also confirmed that biliverdin administration after hemorrhagic shock and resuscitation had protective effects on lung injury. Our findings suggest that biliverdin has a protective role, at least in part, against hemorrhagic shock and resuscitation-induced lung injury through anti-inflammatory and anti-oxidant mechanisms.

## Introduction

Hemorrhagic shock and resuscitation (HSR) causes a systematic inflammatory response that ultimately leads to multiple organ failure.[1], [2] Acute lung injury (ALI) after HSR is a major cause of dysfunction in other organs because of the systemic release of inflammatory mediators.[3] HSR also causes oxidative damage to endothelial cells and involves the accumulation of neutrophils and activation of nuclear factor-kappa B (NF-κB), a transcription factor related to the production of many proinflammatory molecules.[4], [5] This interaction between inflammation and oxidative damage is an important mechanism involved in ALI after HSR.

Bilirubin is considered toxic in high concentrations because it exerts harmful effects on the central nervous system.[6] The anti-oxidant activity of bilirubin was shown in for the first time in the 1980s.[7] It is now well known that the potent anti-oxidant action of bilirubin is amplified by the biliverdin (BV)/bilirubin redox cycle mediated by biliverdin reductase (BVR).[8] BVR converts BV to bilirubin, which is converted back into BV through the actions of reactive oxygen species (ROS). In addition, BV has been shown to play a cytoprotective role in various experimental models of oxidative tissue injury without abnormally elevating serum bilirubin levels.[9]–[13] In particular, it has been already shown that biliverdin administration improves acute lung injury induced by lipopolysaccharide (LPS).[13] However, protective effects of biliverdin administration should be determined in other settings to elucidate its mechanistic details.

In the present study, we administered BV prior to HSR and examined its effects on HSR-induced lung injury in rats. The aim of our study is to examine whether BV ameliorate HSR-induced lung injury by suppressing gene expression of inflammatory mediators and decreasing oxidative DNA damage in the lungs.

## Materials and Methods

### 1. Animals and treatments

The study reported herein conformed to the National Institutes of Health Guidelines for Animal Research (Guide for the Care and Use of Laboratory Animals) and protocol was approved by the Animal Use and Care Committee of Okayama University Medical School (OKU-2012151). Male Sprague–Dawley rats weighing 350 to 400 g were purchased from Clea Japan, Inc. (Tokyo, Japan). The rats were housed in temperature-controlled (25°C) rooms with 12-hr light/ dark cycles and allowed access to water and chow until the start of experiments. All surgical procedures and painful treatments were done under anesthesia with ethyl ether. To decrease stress during hemorrhagic shock and resuscitation, intraperitoneal sodium pentobarbital injection was used. Ethly ether was also used for euthanasia.

### 2. Hemorrhagic shock protocol

Rats were anesthetized with intraperitoneal sodium pentobarbital (50 mg/kg) injections and subjected to sham or HS surgery. In brief, the left femoral artery and vein were dissected using aseptic procedures and catheterized with heparinized polyethylene tubing. The left femoral artery catheter was used to measure blood pressure while the left femoral vein catheter was used to induce HSR. After measuring the baseline blood pressure, hemorrhage was initiated by bleeding into a heparinized syringe (10 units/ml) for a period of 15 min to achieve a mean arterial blood pressure of 30 mmHg. The animals were maintained at this blood pressure (30±5 mmHg) for 60 min by further blood withdrawal or shed blood infusion. The animals were then resuscitated for 15 min by administration of all shed blood until pressure was restored to baseline levels. The sham group was subjected to all procedures except bleeding. The animals were allowed to breathe spontaneously throughout the experiment. All procedures were performed on a heating pad with continuous monitoring and regulation of rectal temperature within physiologic range.

### 3. Doses of BV and serum bilirubin levels

To examine the effects of BV administration on serum bilirubin levels, the rats were administered various doses of BV (0, 15, 35, 70, 100 mg/kg) via the tail vein under light anesthesia with ethyl ether. Serum bilirubin levels were measured at 1 h after BV administration using ABL800FLEX (Radiometer Medical, Copenhagen, Denmark).

### 4. Experimental design

To examine the effects of BV administration on HSR-induced lung injury, rats were randomly divided into the following four groups: a vehicle/sham group (n = 11), which was administered vehicle (saline) before sham surgery, a BV/sham group (n = 11), which was administered BV before sham surgery, a vehicle/HSR group (n = 11), which was administered vehicle before HSR, and a BV/HSR group (n = 11), which was administered BV before HSR. BV (35 mg/kg) or vehicle was injected via the tail vein 1 h before the HS-inducing or sham surgery. At specific timepoints (3 or 12 h) after resuscitation, the animals were euthanized by decapitation under light anesthesia with ethyl ether. The lungs were excised, rinsed quickly and gently in saline, and stained with hematoxylin and eosin and naphthol AS-D chloroacetate esterase for histological analysis and 8-hydroxy-2′ deoxyguanosine (8-OHdG) for immunohistochemical analysis. For the preparation of RNA and the measurement of myeloperoxidase (MPO) activity and 8-OHdG levels, tissue samples were frozen immediately in liquid nitrogen and stored at −80°C until further use.

### 5. BV treatment

BV hydrochloride (Frontier Scientific, Inc., Logan, UT, USA) was dissolved in 0.2 N NaOH and adjusted to pH 7.4 with 1 N HCl. The solution was passed through a 0.45-µm filter (CORNING®) and diluted in saline (35 mg/kg in 1 mL saline) to approximately 20 mM (19.78–22.61) prior to injection. Because BV is light sensitive, the solutions were prepared and the experiments were performed under dim lighting conditions.

### 6. Preparation of cDNA

Template cDNAs for tumor necrosis factor (TNF)-α and inducible nitric oxide synthase (iNOS) were prepared as described previously.[14], [15] All probes used for Northern blot analysis were [α-^32^P] deoxycytidine triphosphate (dCTP) (PerkinElmer Japan Co. Yokohama, Japan)-labeled cDNA prepared using a random primer DNA labeling system (GE Healthcare Japan Co. Tokyo, Japan) according to the manufacturer’s instructions.

### 7. RNA isolation and Northern blot analysis

Rat lungs were excised 3 h after resuscitation, and total RNA was isolated from the lung tissues using Tri-Reagent® (Sigma-Aldrich Japan Co. Tokyo, Japan) according to the manufacturer’s protocol. Total RNA (20 µg) was subjected to electrophoresis in 1.2% (w/v) agarose gel containing 6.5% (v/v) formaldehyde. After blotting on a sheet of Bio-Rad Zeta-Probe membrane (Bio-Rad Laboratories Co. Tokyo, Japan), RNA samples were hybridized with a [α-^32^P] dCTP-labeled cDNA probe and washed under stringent conditions. The membrane was exposed to a sheet of Fuji Medical radiograph film with an intensifying screen at −70°C. Autoradiographs and 18S ribosomal RNA were quantified using an image scanner (GelPrint^™^ 2000i, Genomic Solutions, Inc., Ann Arbor, MI, USA) and computerized image analysis software (Basic Quantifier^™^ Version 3.0, Genomic Solutions, Inc.).

### 8. Histological analysis

For histological examinations, tissues were excised 12 h after resuscitation and fixed in 10% neutral buffered formalin, embedded in paraffin, and sectioned at a thickness of 4–6 µm. After deparaffinization and dehydration, the sections were stained with hematoxylin and eosin and examined by an observer blinded to the treatment using a light microscope. Histopathological scoring analysis was performed according to previously described methods with modifications for five independent experiments.[16]–[18] A total of 10 areas of lung parenchyma from each animal were graded as 0 (no findings or normal), 1 (mild), 2 (moderate), or 3 (severe) for each of the following four parameters: intravascular congestion, pulmonary edema, inflammatory cellular infiltration, and intra-alveolar hemorrhage. The result was expressed as a mean of the sum of individual scores for each of these parameters.

The neutrophils in the lungs were stained with a naphthol AS-D chloroacetate esterase-staining kit (Sigma Diagnostics, St. Louis, MO, USA) using sections adjacent to those used for histopathological analysis.[19] The number of positively stained cells was counted in five nonconsecutive sections per rat at 400× magnification by an observer blinded to the treatment.

### 9. Lung wet-weight to dry-weight (wet/dry) ratio

At 12 h after resuscitation, left lung tissue samples were blotted, weighed, and dried at 110°C for 24 h. The dry tissue weight was then determined and the wet/dry ratio calculated as an index of pulmonary edema.[14], [20], [21]

### 10. Lung MPO assay

Lung MPO activity at 12 h after resuscitation was measured as described by Bradley et al.[22], albeit with some modification.[14] Briefly, the tissue was homogenized in 50 mM potassium phosphate buffer (pH 6.0) containing 0.5% (w/v) hexadecyltrimethylammonium bromide (Nacalai Tesque, Kyoto, Japan), followed by centrifugation at 15,000 *g*. After centrifugation, 0.1 ml of the supernatant was mixed with potassium phosphate buffer (pH 6.0) containing 0.167 mg/ml o-dianisidine dihydrochloride (Sigma-Aldrich Japan Co., Tokyo, Japan). Following the addition of 0.3% hydrogen peroxide to the mixture, the increase in color was monitored at 460 nm for 1 min using a spectrophotometer (U-3000^™^ HITACHI, Tokyo, Japan). The protein concentration of the supernatant was determined using a Pierce® BCA^™^ Protein Assay Kit (Thermo Fisher Scientific, Inc., Rockford, IL, USA) according to the manufacturer’s instructions. Values are reported as change in optical density (ΔOD) per milligram of protein.

### 11. Measurement of lung 8-OHdG levels

Lung samples 3 h after resuscitation were homogenized using Cryo-Press (Microtec Co., Chiba, Japan). Mitochondrial DNA was isolated from the lung homogenates using a DNA extractor kit (Wako, Osaka, Japan). The 8-OHdG levels in the mitochondrial DNA were determined using an enzyme-linked immunosorbent assay kit (High sensitivity; Japan Institute for the Control of Aging, Shizuoka, Japan) according to the manufacturer’s instructions.[23] A total of 20 assays were performed in duplicate. Values are reported as the amount of 8-OHdG (nanogram) per milligram of mitochondrial DNA.

### 12. Immunohistochemistry

Immunohistochemical analysis was performed using the indirect immunofluorescence method. Lung tissues were excised 3 h after resuscitation and fixed in 10% neutral-buffered formalin, embedded in paraffin, and sectioned at a thickness of 4–6 µm. Following antigen retrieval in citrate buffer (0.01 M, pH 6.0) with autoclave heat treatment, nonspecific binding sites were blocked with 5% normal goat serum for 60 min. Slides were incubated overnight at 4°C with a mouse anti-8-OHdG monoclonal antibody (Japan Institute for the Control of Aging) and a rabbit anti-aquaporin 5 polyclonal antibody (Alomone Labs, Ltd., Jerusalem, Israel) at a dilution of 1∶100 in 0.01 M phosphate-buffered saline containing 0.3% Triton X-100. For fluorescent visualization of the bound primary antibody, the slides were further incubated for 90 min with Alexa Flour® 488 goat anti-mouse IgG antibody (Molecular Probes, Inc., Eugene, OR, USA) with a green fluorescent label and Alexa Flour® 555 goat anti-rabbit IgG antibody (Molecular Probes, Inc.) with a red fluorescent label. Normal mouse and rabbit serum were used as controls for nonspecific staining. Images were obtained using a Zeiss confocal laser scanning microscope model LSM510 (Zeiss, Jena, Germany). All images were visualized with a 20×0.5 objective lens.

### 13. Effect of BV administration after HSR

To examine the effects of BV administration after HSR, the rats were randomly divided into two groups: a HSR/Vehicle group (n = 10), which was administered vehicle after HSR, and a HSR/BV group (n = 10), which was administered BV after HSR. BV (35 mg/kg) or vehicle was injected via the femoral vein after resuscitation. We examined histological analysis and 8-OHdG expression in the lungs. Each measurement had been done as described above.

### 14. Statistical analysis

Data are expressed as mean ± standard deviation. Statistical analysis was performed using Student’s t-test or analysis of variance followed by Tukey–Kramer honestly significant difference test as appropriate. Comparisons were examined using JMP®9 software (SAS Institute Inc., Cary, NC, USA). *P*<0.05 was considered statistically significant.

## Results

### 1. Effect of BV adminisitration on serum bilirubin levels

It is well known that BV catalyzes to bilirubin by BVR; therefore, we examined the effects of BV administration at various doses (0, 15, 35, 70, and 100 mg/kg) on serum bilirubin levels. At 1 h after treatment, serum bilirubin levels increased in a dose-dependent manner, confirming that BV was converted to bilirubin promptly after administration as expected. Because the levels of bilirubin generated after the administration of 35 mg/kg BV were elevated but within the normal range of <1 mg/dl, and because BV has been shown to exert protective effects at this dose in models of organ transplantation and endotoxin-induced ALI, [10], [13] we decided to use 35 mg/kg of BV in subsequent studies.

### 2. Effect of BV administration on histological changes in the lungs after HSR

**Figure 1:**
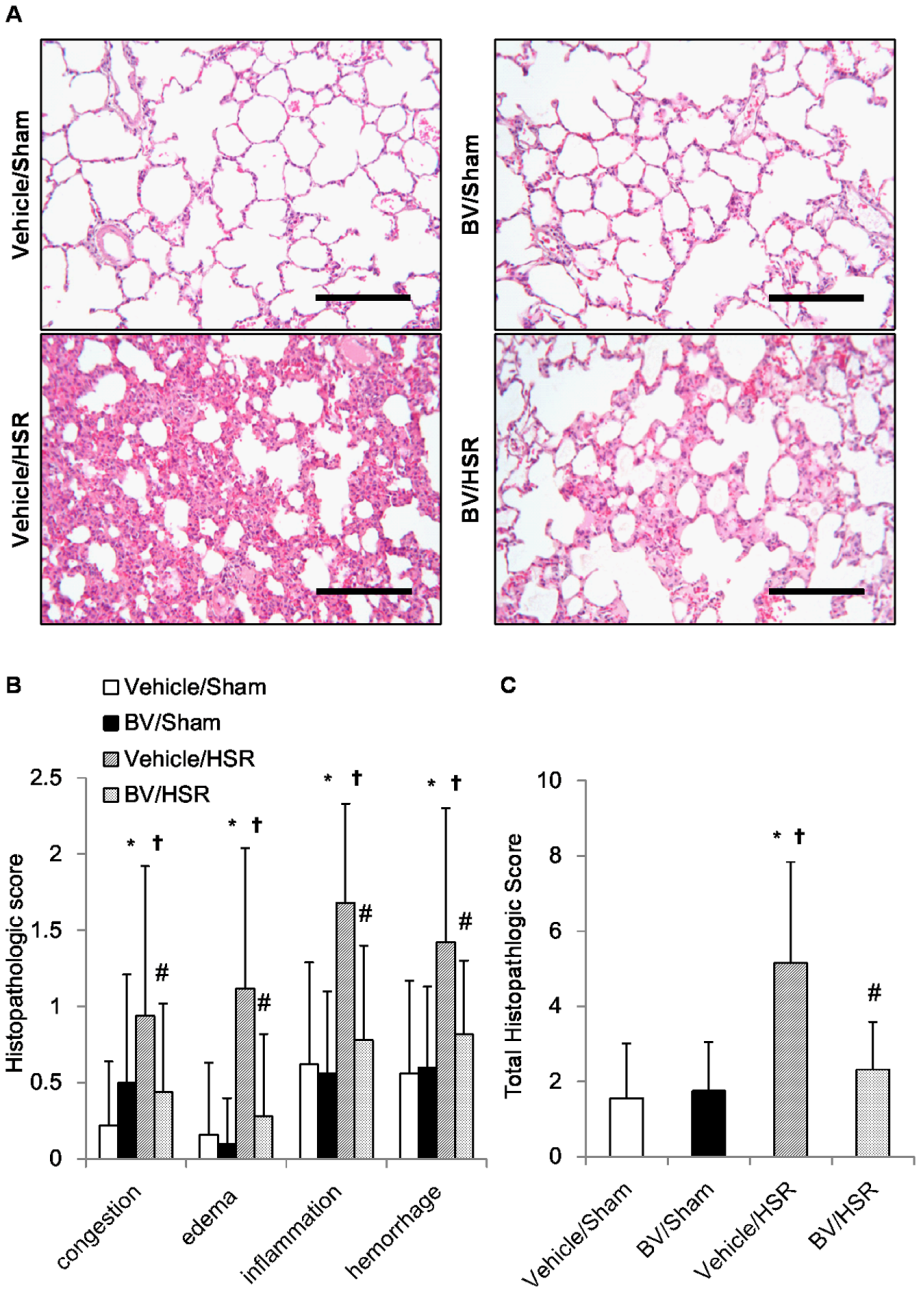
Lung histopathological changes after hemorrhagic shock and resuscitation (HSR). Lungs from the HSR group rats treated with or without biliverdin (BV) administration were excised 12 h after resuscitation and subjected to histological analysis. (**A**) Representative images from five independent experiments (hematoxylin–eosin staining, original magnification ×200, scale bar  = 100 µm). (**B**) The severity of histopathological changes in the lungs was graded for congestion, edema, inflammation, and hemorrhage. A total of 10 areas of lung parenchyma from each rat were graded as 0 (no findings or normal), 1 (mild), 2 (moderate), or 3 (severe) for each of the four parameters. (**C**) The sum of histopathological scores for the four parameters were calculated (n = 5 per group). Data are presented as means ± standard deviation and were statistically evaluated using analysis of variance followed by Tukey–Kramer honestly significant difference test. ^*^p<0.01 vs. vehicle/sham; ^†^p<0.01 vs. BV/sham; ^#^p<0.01 vs. vehicle/HSR. Vehicle/sham, vehicle-administered animals subjected to sham surgery; BV/sham, BV-administered animals subjected to sham surgery; vehicle/HSR, vehicle-administered animals subjected to HSR; BV/HSR, BV-administered animals subjected to HSR.

Next we examined the effects of BV administration prior to HS induction on HSR-induced lung injury. Histopathological analysis indicated that the vehicle/HSR group developed interstitial edema as identified by pronounced alveolar septal thickening with marked infiltration of inflammatory cells 12 h after HSR. However, sections from the sham groups appeared normal (Fig. 1, A). In contrast, BV administration prior to HSR obviously mitigated these pathological changes, including congestion, edema, inflammation, and hemorrhage (Fig. 1, A and B). The significant effects of BV were also confirmed by the scoring of histopathologic changes by an independent researcher blinded to the treatment. BV administration greatly suppressed lung injury as evidenced by decreased histopathological damage confirmed by a decrease in the total histopathologic score (Fig. 1, C).

### 3. Effect of BV administration on neutrophil accumulation in the lungs after HSR

**Figure 2:**
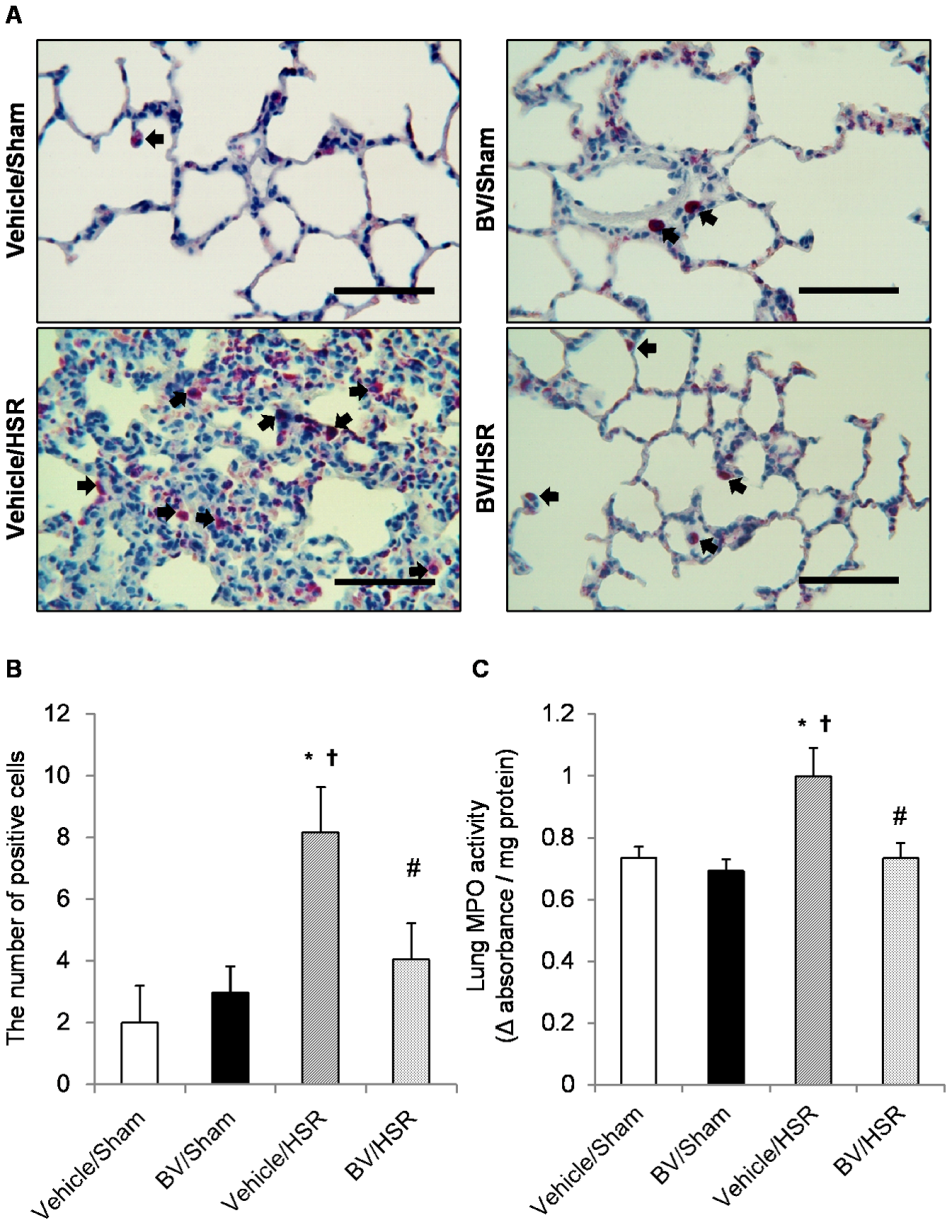
Neutrophil accumulation and myeloperoxidase (MPO) activity in the lungs after hemorrhagic shock and resuscitation (HSR). Lungs from the HSR group rats treated with or without biliverdin (BV) were excised 12 h after resuscitation. Neutrophils were stained by the naphthol AS-D chloroacetate method and MPO activity was measured. (A) Each photograph is representative of five independent experiments. The arrows indicate positively stained neutrophils (original magnification ×400, scale bar  = 50 µm). (B) The number of neutrophils in five nonconsecutive lung sections per rat at a magnification of ×400 (n = 5 per group). (C) Lung MPO activity (n = 6 per group). Data are presented as means ± standard deviation and were statistically evaluated using analysis of variance followed by Tukey–Kramer honestly significant difference test. ^*^p<0.05 vs. vehicle/sham; ^†^p<0.05 vs. BV/sham; ^#^p<0.05 vs. vehicle/HSR. Vehicle/sham, vehicle-administered animals subjected to sham surgery; BV/sham, BV-administered animals subjected to sham surgery; vehicle/HSR, vehicle-administered animals subjected to HSR; BV/HSR, BV-administered animals subjected to HSR.

On the basis of the central role of neutrophils in the pathogenesis of lung injury, we evaluated MPO activity accompanied by neutrophil sequestration. Pulmonary MPO activity was markedly elevated in the vehicle/HSR group 12 h after resuscitation compared with that in the sham groups (Fig. 2, C). BV treatment before HSR significantly decreased MPO activity in the lungs compared with vehicle treatment before HSR (Fig. 2, C). Consistent with changes in MPO activity, the number of infiltrating neutrophils in the lungs also markedly increased in the vehicle/HSR group at the same timepoint (Fig. 2, A and B). In contrast, neutrophil recruitment in the lungs was markedly decreased in the BV/HSR group, with a marked decrease in number (Fig. 2, A and B).

### 4. Effect of BV administration on lung edema after HSR

**Figure 3:**
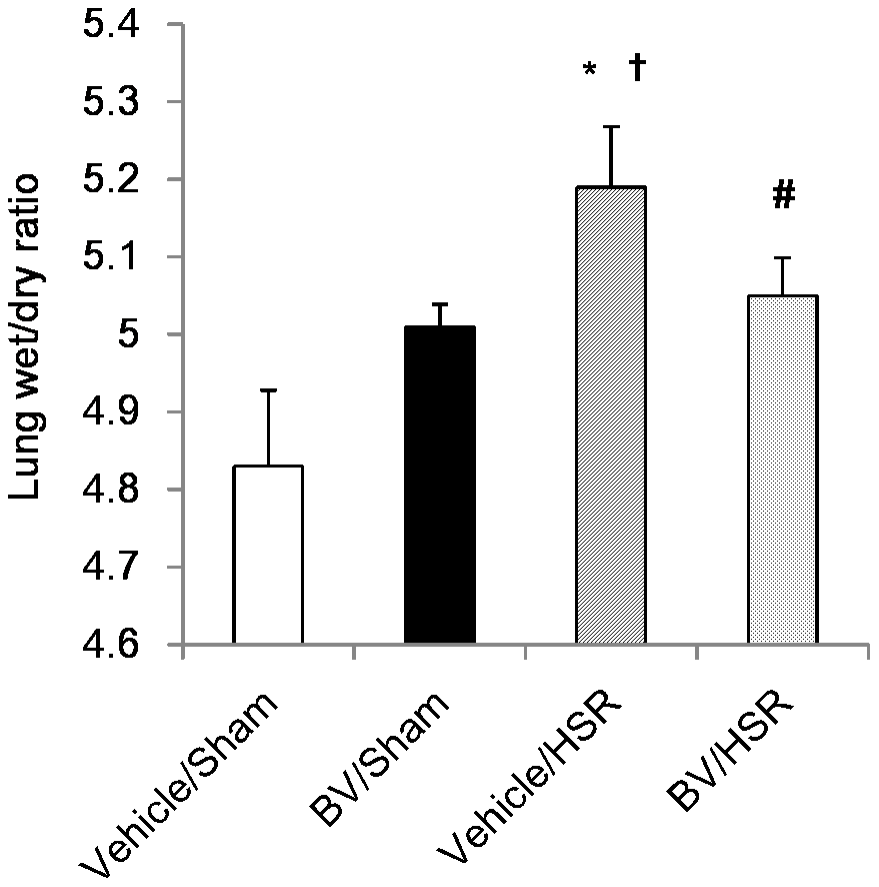
Lung wet/dry ratio after hemorrhagic shock and resuscitation (HSR). Lungs from the HSR group rats treated with or without biliverdin (BV) were excised 12 h after resuscitation and lung wet/dry ratio was measured. Data are presented as means ± standard deviation (n = 5 per group). Statistical analysis was performed using analysis of variance followed by Tukey-Kramer honestly significant difference test. ^*^ p<0.05 vs. vehicle/sham; ^?^p<0.05 vs. BV/sham; ^#^p<0.05 vs. vehicle/HSR. Vehicle/sham, vehicle-administered animals subjected to sham surgery; BV/sham, BV-administered animals subjected to sham surgery; vehicle/HSR, vehicle-administered animals subjected to sham; BV/HSR, BV-administered animals subjected to sham.

ALI is characterized by pulmonary edema caused by alveolar and interstitial fluid accumulation, resulting in a decrease in lung compliance and hypoxemia.[23] Lung wet/dry ratio, a parameter of lung edema, significantly increased 12 h in the HSR groups compared with that in the sham groups (Fig. 3). However, BV administration significantly attenuated HSR-induced lung edema (Fig. 3).

### 5. Effect of BV administration on gene expression of the HSR-induced inflammatory mediators TNF-α and iNOS

**Figure 4:**
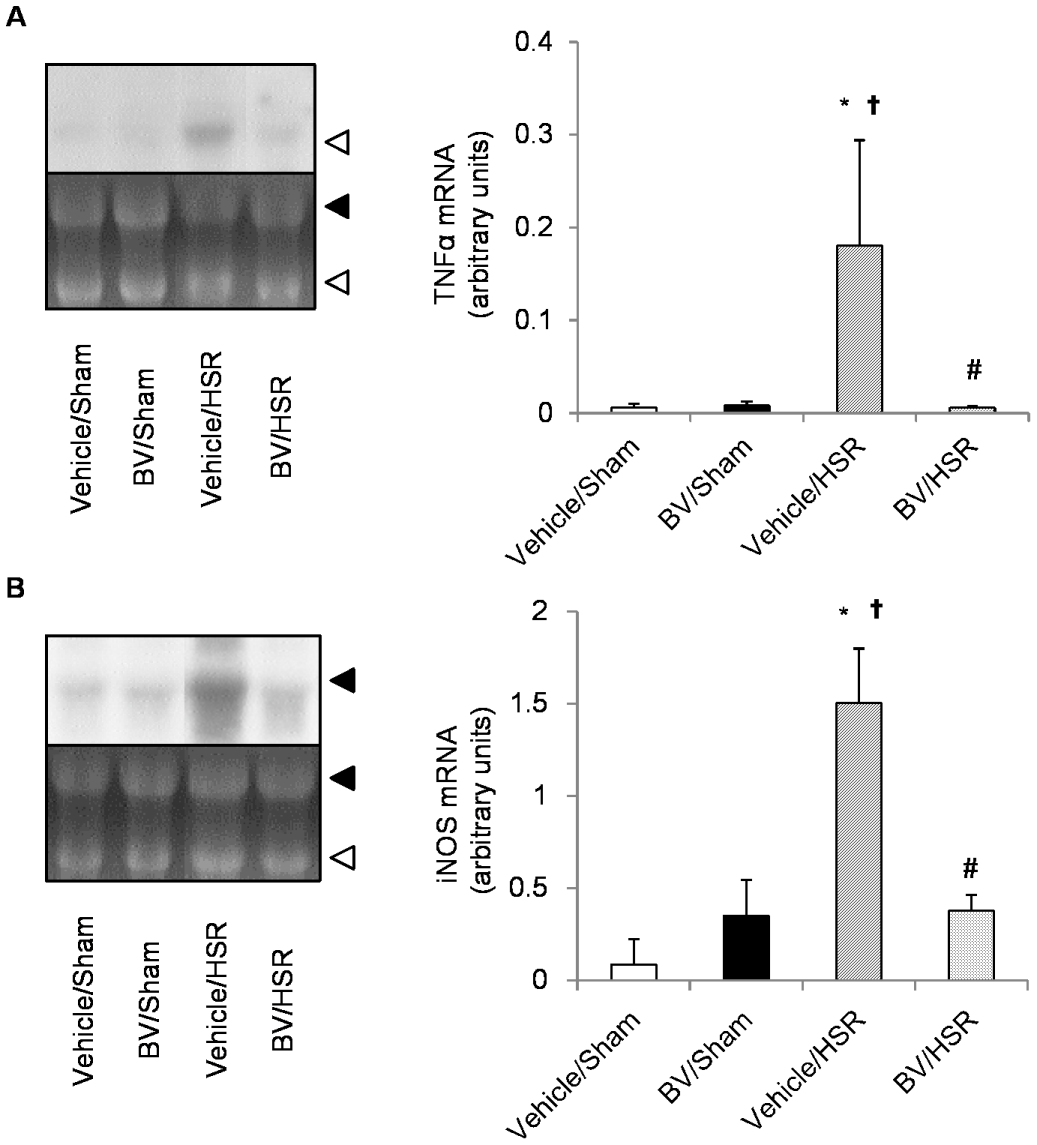
Gene expression of inflammatory mediators in the lungs after hemorrhagic shock and resuscitation (HSR). Lungs from the HSR group rats treated with vehicle or biliverdin (BV) were excised at 3 h after resuscitation and the levels of tumor necrosis factor (TNF)-α and inducible nitric oxide synthase (iNOS) mRNA were determined by Northern blot analysis. (Left) The autoradiographic signals of RNA blot hybridized with [α-^32^P] deoxycytidine triphosphate-labeled TNF-α (**A**) or iNOS (**B**) cDNA. (Right) Concentrations of TNF-α and iNOS mRNA were expressed as arbitrary units. Data are presented as means ± standard deviation and were statistically evaluated using analysis of variance followed by Tukey–Kramer honestly significant difference test (n = 3 per group). ^*^p<0.05 vs. vehicle/sham; ^†^p<0.05 vs. BV/sham; ^#^p<0.05 vs. vehicle/HSR. Vehicle/sham, vehicle-administered animals subjected to sham surgery; BV/Sham, BV-administered animals subjected to sham surgery; vehicle/HSR, vehicle-administered animals subjected to HSR; BV/HSR, BV-administered animals subjected to HSR.

To elucidate the molecular mechanism underlying the anti-inflammatory effects of BV, we examined the effects of BV treatment on the gene expression of the inflammatory mediators such as TNF-α and iNOS using Northern blot analysis. Although mRNA levels of TNF-α and iNOS were barely detectable in the sham groups irrespective of the presence or absence of BV, these genes were significantly upregulated in the vehicle/HSR group 3 h after resuscitation (Fig. 4). The mRNA levels of TNF-α and iNOS were significantly decreased in the BV/HSR group compared with those in the vehicle/HSR group (Fig. 4).

### 6. Effect of BV administration on lung 8-OHdG expression

**Figure 5:**
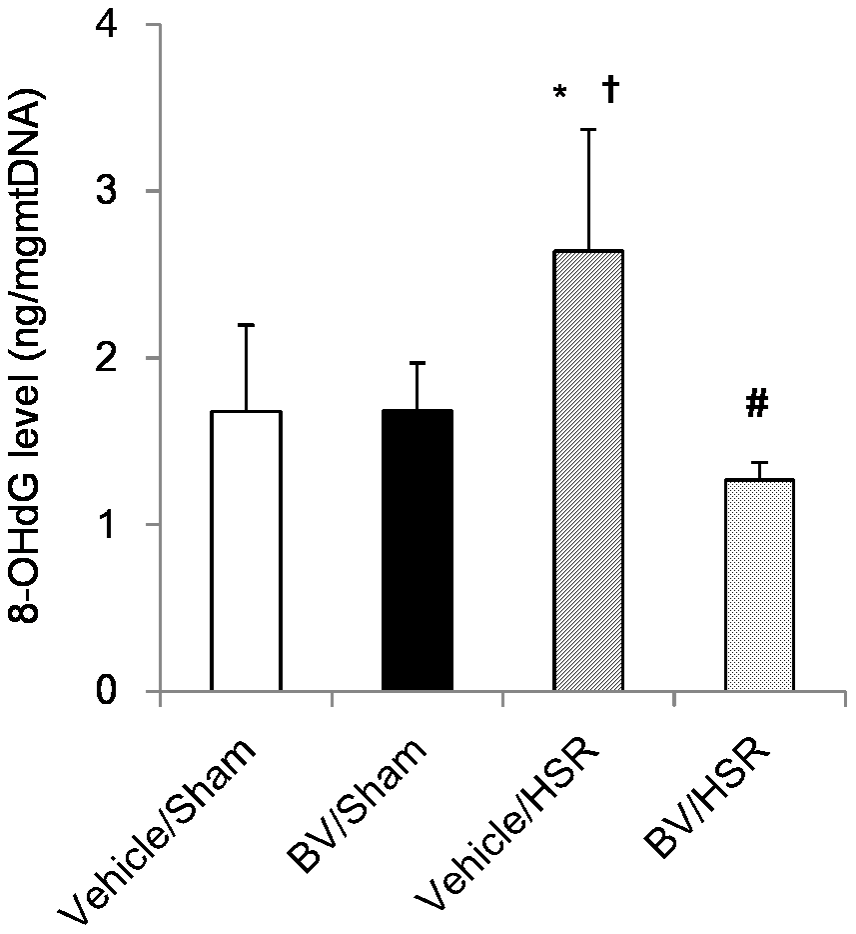
Levels of 8-hydroxy-2′ deoxyguanosine (8-OHdG) in the lungs after hemorrhagic shock and resuscitation (HSR). Lungs from the HSR group rats treated with or without biliverdin (BV) were excised 3 h after resuscitation and mitochondrial 8-OHdG levels were measured. Data are presented as means ± standard deviation (n = 5 per group). Statistical analysis was performed using analysis of variance followed by Tukey–Kramer honestly significant difference test. ^*^p<0.05 vs. vehicle/sham; ^†^p<0.05 vs. BV/sham; ^#^p<0.05 vs. vehicle/HSR. Vehicle/sham, vehicle-administered animals subjected to sham surgery; BV/sham, BV-administered animals subjected to sham surgery; vehicle/HSR, vehicle-administered animals subjected to HSR; BV/HSR, BV-administered animals subjected to HSR.

**Figure 6:**
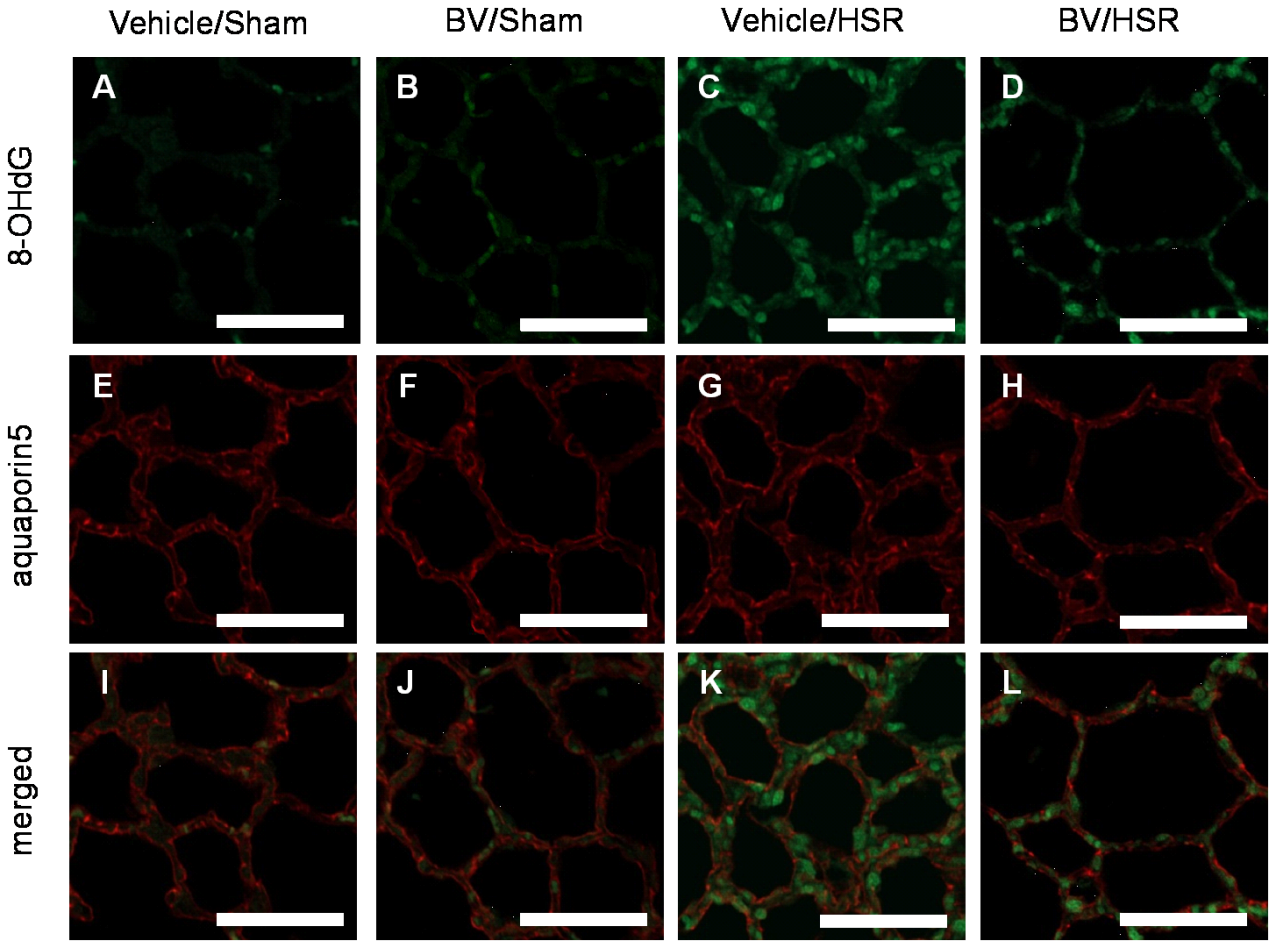
Aquaporin-5 and 8-hydroxy-2′ deoxyguanosine (8-OHdG) expression in the lungs after hemorrhagic shock and resuscitation (HSR). Sections of lungs obtained 3 h after resuscitation were subjected to fluorescent immunohistochemical analysis for 8-OHdG (green) and aquaporin-5 (red). Above row: 8-OHdG-positive cells in the vehicle/sham (A), BV/sham (B), vehicle/HSR (C) and BV/HSR groups (D). Middle row: aquaporin-5-positive cells in the vehicle/sham (E), BV/HSR (F), vehicle/HSR (G), and BV/HSR groups (H). Bottom row: merged images of 8-OHdG and aquaporin-5 in the vehicle/sham (I), BV/sham (J), vehicle/HSR (K), and BV/HSR groups (L). Vehicle/sham, vehicle-administered animals subjected to sham surgery; BV/Sham, BV-administered animals subjected to sham surgery; vehicle/HSR, vehicle-administered animals subjected to HSR; BV/HSR, BV-administered animals subjected to HSR. Scale bar  = 50 µm. All images shown were visualized with a 20×0.5 objective lens.

To examine oxidative DNA damage, we measured 8-OHdG levels in lung tissue and performed immunohistochemical analysis. HSR increased mitochondrial 8-OHdG levels in the lung by 1.5-fold compared with sham surgery (Fig. 5). BV administration before HSR significantly decreased mitochondrial 8-OHdG levels to almost the same level as that in the sham groups (Fig. 5). According to immunohistochemistry, 8-OHdG-positive cells were negligibly detected in the sham groups (Fig. 6A and I). However, strong positive signals (green color) for 8-OHdG were predominantly observed in the pulmonary interstitium of the HSR groups (Fig. 6C and K). In contrast, BV administration quenched 8-OHdG signals in the lungs of animals in the HSR groups (Fig. 6D and L). These results revealed that BV administration significantly inhibited HSR-induced oxidative DNA damage.

### 7. Effect of BV administration after HSR

**Figure 7:**
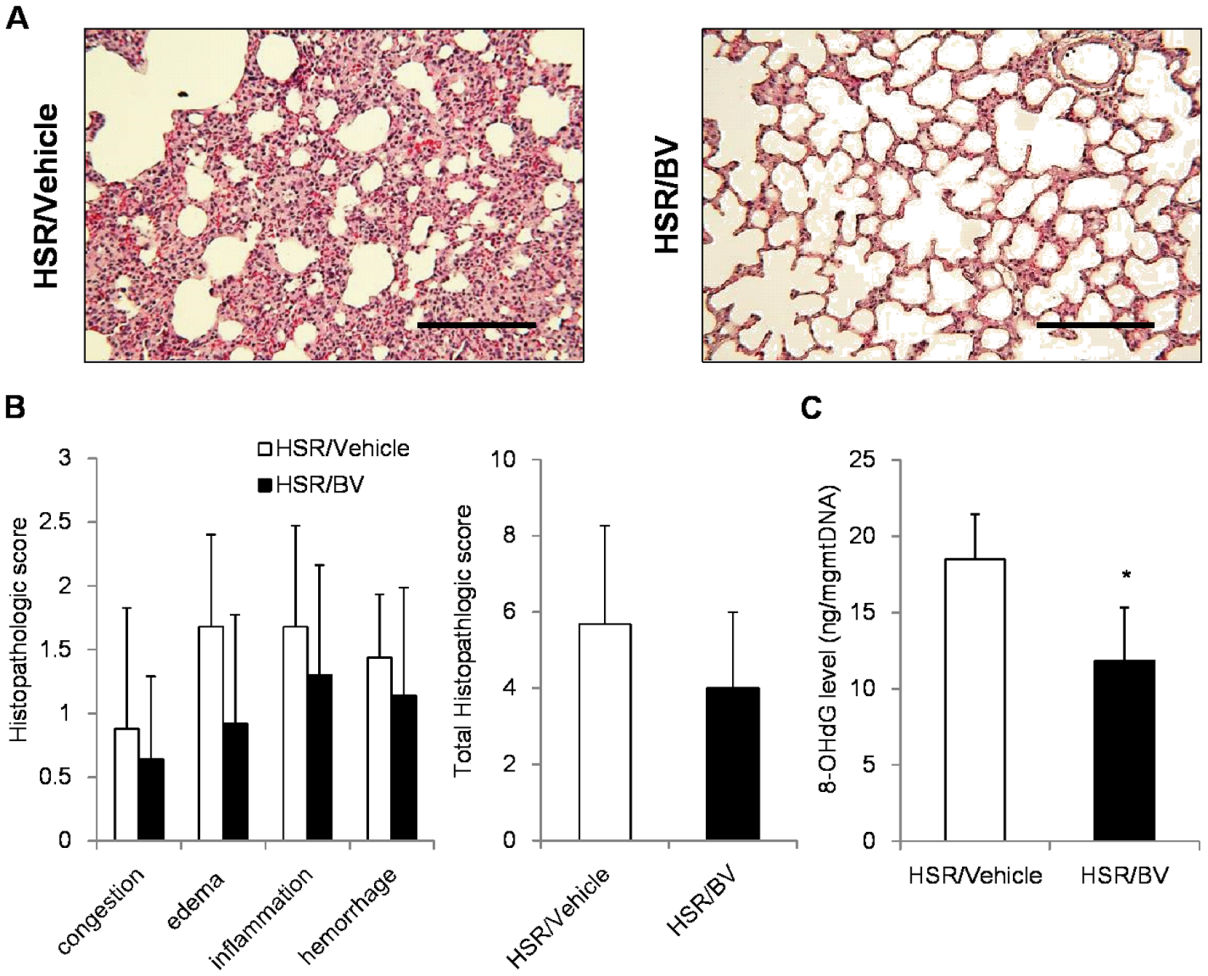
Effect of BV administration after hemorrhagic shock and resuscitation (HSR) in the lungs. The rats were randomly divided into two groups: a HSR/Vehicle group, which was administered vehicle after HSR, and a HSR/BV group, which was administered BV after HSR. BV (35 mg/kg) or vehicle was injected via the femoral vein after resuscitation. (**A**) Representative images from five independent experiments (hematoxylin–eosin staining, original magnification ×200, scale bar  = 100 µm). (**B**) lung histopathological score 12 h after HSR. (**C**) Mitochondrial 8-OHdG levels in the lungs 3 h after HSR. Data are presented as means ± standard deviation (n = 5 per group). Statistical analysis was performed using Student’s t-test. ^*^p<0.05 vs. HSR/Vehicle.

To enhance clinical significance of our findings, we assessed the protective effects of BV administration after resuscitation. In histological examinations, HSR-induced lung injury appeared to be improved by BV administration (Fig. 7A), although the difference in the histopathological score did not reach statistical significance (Fig. 7B). However, mitochondrial 8-OHdG, a marker DNA oxidative damage, was significantly reduced by the administration of BV even after HSR (Fig. 7C).

## Discussion

This study demonstrated that 35 mg/kg of BV administered intravenously before HSR significantly ameliorated HSR-induced lung injury. This fact was confirmed by improvements in histological changes, neutrophil infiltration, and lung edema. We also found that BV administration significantly decreased the gene expression of inflammatory mediators such as TNF-α and iNOS. Finally, we found that BV administration decreased the degree of DNA fragmentation due to oxidative stress by assessing lung 8-OHdG levels using an enzyme-linked immunosorbent assay and immunohistochemistry. These findings indicated that BV has the potential to protect lungs from HSR-induced injury through its anti-inflammatory and anti-oxidative properties.

In this study, we demonstrated that BV administration prior to HSR improved histopathological changes in the lungs. Previous studies showed that BV treatment significantly decreased the infiltration of neutrophils and inflammatory macrophages and ameliorated tissue injury in models of hepatic[9], cardiac[10], and small intestine ischemia–reperfusion (I/R) injury.[11] BV administration also suppressed monocyte and polymorphonuclear leukocyte accumulation in bronchoalveolar lavage fluid and ameliorated endotoxin-induced lung injury.[13] Of note, our study, for the first time, determined that BV administration has a protective role in ALI after HSR. Considering our results and those from other reports [9]-[11], it is very plausible that BV can protect various organs, particularly the lungs, from I/R injuries.

On exploring the mechanisms underlying the protective effects of BV administration on ALI after HSR, we found that BV administration suppressed HSR-induced gene expression of inflammatory mediators such as TNF-α and iNOS. This finding is consistent with that of previous reports.?Constantino et al. showed that BV therapy downregulated the expression of the same inflammatory mediators in a hepatic I/R model.[9] Nakao et al. also showed that small intestine transplantation in the rat significantly increased iNOS and COX-2 mRNA while BV injections decreased the increased expression of these inflammatory mediators by almost half.[11] Moreover, BV treatment decreased serum proinflammatory cytokines and increased serum anti-inflammatory cytokines in a lipopolysaccharide-induced ALI model.[13] Our study results suggest that BV administration decreased inflammatory gene expression in an HSR-induced ALI model. Therefore, BV administration definitely has anti-inflammatory effects, and as such, this characteristic is an important mechanism responsible for its protective properties with regard to lung injury.

This study also demonstrated that BV exerted anti-oxidant effects on HSR-induced oxidative DNA damage of the lung. 8-OHdG is produced when the guanine in DNA suffers from oxidative damage due to ROS and lipid peroxide.[24] Measurement of 8-OHdG is a reliable indicator of oxidative damage in several tissues.[23], [25] Previous studies suggested that BV has strong anti-oxidant potential *in vitro*.[7] However, there are few studies showing the physiological anti-oxidant effects of BV. In an intestinal I/R model, BV elevated the intestinal reductive capacity and decreased tissue malondialdehyde concentrations.[11] In an oxidative brain injury model, BV significantly decreased cerebral infarct size and prevented lipid oxidation and oxidative DNA damage.[12] Our study also demonstrated the anti-oxidant potential of BV on ALI induced by HSR. These results indicate that BV has a strong protective effect on oxidative damage in various organs. Although we showed that BV had anti-inflammatory and anti-oxidant effects on HSR-induced lung injury in this study, the detailed molecular mechanisms underlying these effects remain elusive.

Our study has several limitations. First, we did not measure NF-κB, a transcriptional factor for inflammatory mediators; however, previous studies demonstrated that BV administration suppressed NF-κB activation and led to cytoprotection.[11], [13] We hypothesized that the anti-inflammatory potential of BV may be mediated by this effect on NF-κB. Second, we also did not measure the exact levels of ROS induced by HSR. However, ROS levels are difficult to examine because of instability. Actually, most studies on the anti-oxidant effects of BV proved that BV treatment decreased the metabolites induced by ROS.[11], [12] Third, we used shed blood to resuscitate hemorrhagic shock. This blood transfusion itself could cause lung injury. In a recent report, however, it has been shown that transfusion of older blood can lead to organ damage and to lung injury.[26], [27] These studies also confirmed that transfusion of new blood do not induce severe organ damages. We used their own shed blood within an hour. It is unlikely that this procedure cause such a severe lung injury as shown our model. Finally, we did not assess BVR activities in our study. Previous studies suggested that BVR had an important role in the redox cycle of oxidative damage.[8], [28] The protective role of BV administration would probably be affected by its interaction with BVR; however, there is no definite evidence of BV–BVR interaction *in vivo*, and we consider that it would be an important target for future research.

We believe that BV is more suitable for clinical use than bilirubin because BV is water soluble, readily excreted, and nontoxic. BV is converted to bilirubin by BVR in mammalian tissue, and hyperbilirubinemia is associated with a risk of neurological deficit.[6] The 35-mg/kg dose of BV elevated serum bilirubin levels 1 h after treatment, although the elevated level was not outside the high normal range of <1 mg/dl in this study. Moreover, BV treatment itself had no influence on histopathological changes and the gene expression of inflammatory mediators compared with vehicle treatment.

We also examined the protective effect of BV after HSR on ALI in an additional study. As a result, BV administration after HSR had an anti-oxidant effect and tended to improve lung injury in histological analysis. However, more detailed mechanistic and clinical examinations would be needed to clarify its role in clinical settings.

In conclusion, we reported that HSR causes significant tissue inflammation as evidenced by the increase in gene expression of inflammatory mediators, neutrophil migration, and pulmonary edema. BV administered prior to HSR significantly decreased HSR-induced oxidative DNA damage and subsequently ameliorated HSR-induced lung injury. These findings indicate that BV suppresses HSR-induced lung injury, at least in part, through anti-inflammatory and anti-oxidant mechanisms.
